# Genetic Variations in *ABCG2* Gene Predict Breast Carcinoma Susceptibility and Clinical Outcomes after Treatment with Anthracycline-Based Chemotherapy

**DOI:** 10.1155/2015/279109

**Published:** 2015-11-08

**Authors:** Huizhe Wu, Yong Liu, Hui Kang, Qinghuan Xiao, Weifan Yao, Haishan Zhao, Enhua Wang, Minjie Wei

**Affiliations:** ^1^Department of Pharmacology, School of Pharmacy, China Medical University, Shenyang, Liaoning 110122, China; ^2^Department of Clinical Laboratory, Shengjing Hospital of China Medical University, Heping Ward, Shenyang, Liaoning 110003, China; ^3^Department of Clinical Laboratory, The First Hospital of China Medical University, Heping Ward, Shenyang, Liaoning 110001, China; ^4^Institute of Pathology and Pathophysiology, The First Hospital and College of Basic Medical Sciences of China Medical University, Heping Ward, Shenyang, Liaoning 110001, China

## Abstract

The genetic variants of the ATP-binding cassette, subfamily G, member 2 (*ABCG2*) are known to be involved in developing cancer risk and interindividual differences in chemotherapeutic response. The polymorphisms in* ABCG2* gene were genotyped by using PCR-RFLP assays. We found that* ABCG2* G34A GA/AA genotype, C421A AA genotype, and haplotypes 34A-421C and 34G-421A were significantly associated with increased risk for developing breast carcinoma. Furthermore,* ABCG2* C421A AA homozygote had a significant enhanced therapeutic response in patients with neoadjuvant anthracycline-based chemotherapy. Moreover,* ABCG2* G34A AA genotype carriers displayed a longer OS in ER positive patients or PR positive patients after postoperative anthracycline-based chemotherapy. These results suggested that the *ABCG2* polymorphisms might be a candidate pharmacogenomic factor to assess susceptibility and prognosis for breast carcinoma patients.

## 1. Introduction

Breast carcinoma (BC) is one of the most common malignant cancers worldwide and is the leading cause of cancer-related deaths in women [1]. Chemotherapies are often used as neoadjuvant and adjuvant therapy for BC. However, the development of multidrug resistance is a big problem in BC. It is well known that the multidrug resistance involving drug efflux pump systems contributes to chemotherapy failure and poor prognosis in BC patients [2, 3]. ATP-binding cassette (ABC) transporters, which transport a variety of molecules including chemotherapeutic drugs, are known to mediate the multidrug resistance of BC [4–6]. ABCG2 protein, also called breast cancer resistance protein (BCRP), belongs to the family of ABC transporters, mediating high levels of resistance to a variety of anticancer agents [7–9].

The* ABCG2* gene is located on chromosome 4q22 and encodes a 655-amino acid protein. Single-nucleotide polymorphisms (SNPs) in the* ABCG2* gene have been identified in various ethnic populations [10–13]. The most frequent SNPs in the* ABCG2* gene are G34A (rs2231137, V12M), which codes for Val12Met, and C421A (rs2231142, Q141K), which codes for Gln141Lys. These polymorphisms are associated with decreased expression and then reduce transporter activity of the ABCG2 protein [14–17]. For example, the variation of C421A in the* ABCG2* gene is associated with an elevated risk of gout and has been reported to reduce the plasma membrane expression and the transport function of BCRP protein in the model cells [16]. In addition, the ABCG2 protein level in human placenta carrying C421A A allele is significantly lower than that carrying C421A C allele [17]. Therefore,* ABCG2* SNPs alter the expression and transporter activity of ABCG2 protein and thus may be associated with the development of carcinoma and interindividual variability in drug response to anticancer agents and the clinical outcome.

To date, only few studies have investigated the association between the* ABCG2* polymorphisms and the susceptibility and survival of carcinoma [18–23]. In a case-control study, Korenaga et al. found that carrying C421A CC genotype showed an increased risk of developing nonpapillary renal cell carcinoma [18]. Furthermore, Hahn et al. demonstrated that hormone-refractory prostate cancer patients carrying* ABCG2* C421A genotype more likely survived beyond 15 months compared with those carrying C421A CC genotype [19]. In addition, Han et al. reported that the C421A variant was associated with an increased risk for diffuse large B-cell lymphoma (DLBCL), and the C421A CC genotype was associated with poor survival of DLBCL in patients younger than 50 years at diagnosis or with bulky tumor [20]. This study further demonstrated that carriers of G34A AA genotype allele displayed worse survival as a prognostic indicator [20]. Although the associations between* ABCG2* gene polymorphisms and carcinoma risk and prognosis have been evaluated for these carcinomas, the genetic effect of the* ABCG2* polymorphisms on the susceptibility and prognosis of BC is still unclear.

Therefore, in this large prospective cohort, we investigated the G34A and C421A polymorphisms in the* ABCG2* gene between 1169 BC patients and 1244 healthy controls and attempted to explore the correlation between these polymorphisms with BC susceptibility, development, and clinical outcomes after chemotherapy. Our study provides theoretic basis data for personalized BC chemotherapy.

## 2. Materials and Methods

### 2.1. Study Population

In this study, 1230 patients with newly diagnosed BC were recruited consecutively from 2001 to 2012 at the First Hospital of China Medical University (Shenyang, China) and Shengjing Hospital of China Medical University (Shenyang, China). The principal clinical characteristics, including age at diagnosis, gender, menopausal status, and first-degree family history of cancer, were obtained from the interviewer-administered health risk questionnaires and medical records. According to clinical stages, the samples were dichotomized into stages I and II and stages III and IV. Approximately 95% of contacted patients consented to enrollment in the study. We excluded patients, whose blood samples for the* ABCG2* genotyping were not available. Finally, 1169 patients were included in the study.

A clinical oncologist retrospectively collected clinical and pathological characteristics and therapeutic responses after chemotherapy from medical records. Overall, the majority of the patients received anthracycline-based chemotherapy (preoperative neoadjuvant therapy, *n* = 148; postoperative adjuvant chemotherapy, *n* = 761). Clinical tumor response was assessed after the 2nd cycle of first-line preoperative neoadjuvant chemotherapy as complete remission (CR), partial remission (PR), stable disease (SD), or progressive disease (PD) based on the Response Evaluation Criteria in Solid Tumors (RECIST). The anthracycline-based chemotherapy regimens contain CE (Cyclophosphamide and Epirubicin), CA (Cyclophosphamide and Adriamycin), CEF (Cyclophosphamide, Epirubicin, and 5-Fluorouracil), and CAF (Cyclophosphamide, Adriamycin, and 5-Fluorouracil).

In this study, we recruited 1244 unrelated healthy controls matched by gender, age, and menopausal status. The controls had no known medical illness or hereditary disorders and were not taking any medications. Before its commencement, the study was approved by the Research Ethics Committee of China Medical University, and informed consent was obtained from each participant.

### 2.2. Genotyping Analysis

**Figure 1:**
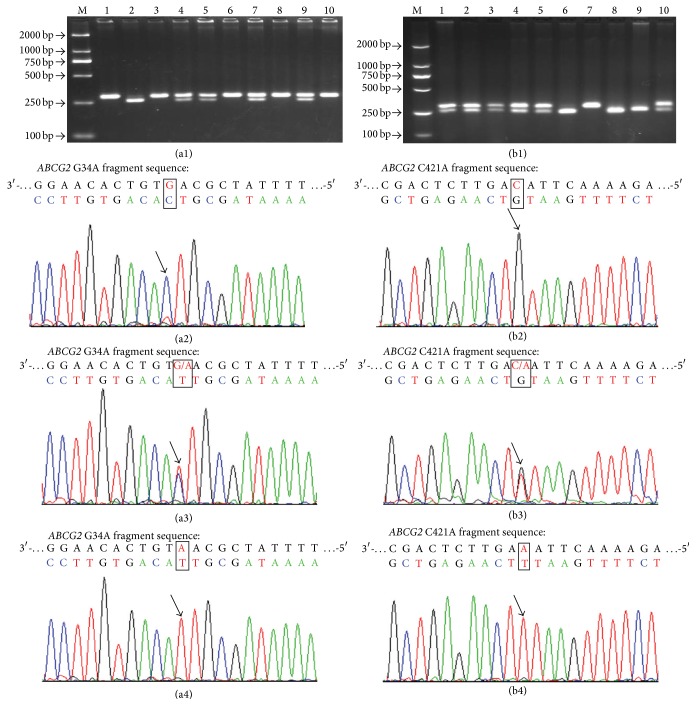
Electrophoretic patterns and DNA sequencing identification for* ABCG2* G34A, C421A polymorphisms. (a1) Representative PCR-RFLP assay for different genotypes containing* ABCG2* G34A polymorphism site: GG genotype (lanes 1, 3, 6, 8, and 10), GA genotype (lanes 4, 5, 7, and 9), AA genotype (lane 2), and D2000DNA ladder marker (lane M); (a2) G34A GG genotype; (a3) G34A GA genotype; (a4) G34A AA genotype. (b1) Representative PCR-RFLP assay for different genotypes containing* ABCG2* C421A polymorphism site: CC genotype (lane 3), CA genotype (lanes 1, 4, and 5), AA genotype (lanes 2 and 6), and D2000DNA ladder marker (lane M); (b2) C421A CC genotype; (b3) C421A CA genotype; (b4) C421A AA genotype.

Genomic DNA was isolated from a leukocyte cell pellet of each blood sample according to the TIANGEN manufacturer's instructions.* ABCG2* polymorphisms were genotyped by polymerase chain reaction-restriction fragment length polymorphism (PCR-RFLP) method. PCR amplification was performed as follows: 100 ng of genomic DNA, 300 nM of each primer, 200 nM dNTPs, and 0.5 U Taq polymerase in PCR buffer (TaKaRa Biotechnology (Dalian) Co. Ltd., Dalian, China). The reaction for amplification was carried out in the following conditions: an initial melting step of 5 min at 94°C, followed by 35 cycles of 30 s at 94°C, 30 s at 58°C, and 1 min at 72°C and a final elongation of 5 min at 72°C. The primers were as follows: (1)* ABCG2* G34A: (forward) 5′-AAAT GTTCATAG CCAGTTTCTTGGA-3′ and (reverse) 5′-ACAGTAATGTCGAAGTTTTTA TCGCA-3′; (2)* ABCG2* C421A: (forward) 5′-GTTGTGATGGGCACTCTGATGGT-3′ and (reverse) 5′-CAAGCCACTTTT CTCA TTGTT-3′. For* ABCG2* G34A, the 291-base pair (bp) PCR products were digested with BseMI (Fermentas Life Science (Beijing) Ltd., Beijing, China) at 55°C overnight. The G allele was uncut, and the A allele was cut into 261 bp and 30 bp bands (Figure 1(a1)). For* ABCG2* C421A, the 302 bp PCR products were digested with TaaI (Fermentas Life Science (Beijing) Ltd., Beijing, China) at 65°C overnight. The C allele was uncut, and A allele was cut into 252 bp and 50 bp bands (Figure 1(b1)). Samples were coded for case-control status, and at least 10% of the samples were randomly selected and subjected to repeat analysis as quality control for verification of genotyping procedures, and some samples were also identified by DNA sequencing analysis (Figures 1(a2~a4) and 1(b2~b4)). Two researchers independently reviewed all genotyping results.

### 2.3. Statistical Analysis

SPSS software package (Statistical Package for the Social Sciences, version 16.0, SPSS Inc., IL, USA) was used to perform statistical analyses. The population genetic analysis program SNPAlyze 2.2 (Dynacom Co. Ltd., Yokohama, Japan) based on the expectation-maximization was used for linkage disequilibrium analysis, haplotype inference, and the Hardy-Weinberg equilibrium test. Statistical significance was set at *P* < 0.05, and all tests were two-sided. The differences in distributions of demographic, epidemiologic, and clinical variables as well as genotypes between the two groups were assessed using *χ* ^2^ test or Fisher exact test. The associations between genotypes and BC risk were assessed using odds ratios (ORs) and 95% CIs from both univariate and multivariate logistic regression analyses. Multivariate Cox proportional hazards regression models were performed to obtain the adjusted hazard ratio (HR) and 95% CI for potential prognostic factors in BC patients. The Kaplan-Meier method and the Log-rank test were used to analyze the associations of the survival time with demographic characteristics and SNPs. The disease-free survival (DFS) was calculated as the time between the first day of treatment and an occurrence of recurrence, metastases, death, or last known follow-up. The overall survival (OS) was calculated as the time between the first day of treatment and death or last known follow-up.

## 3. Results

### 3.1. Demographic and Baseline Characteristics of the Study Population

The present study included 1169 female patients with pathologically confirmed BC and 1244 age- and gender-matched healthy controls. The variables of the cases and controls are summarized in Supplementary Table 1 (Table S1 in Supplementary Material available online at http://dx.doi.org/10.1155/2015/279109). There were no significant differences in the distributions of age (*P* = 0.953) and menopausal status (*P* = 0.321) between cases and controls. The age was matched between cases (range, 22–85 years; median, 50 years) and controls (range, 23–70 years; median, 48 years). Most BC patients were in stage I or II (57.3%), had invasive ductal cancer (IDC) of breast origin (79.9%), and underwent anthracycline-based chemotherapy (65.1%). 54.1% of the patients in this cohort had lymph node metastasis. The variables of age and menopausal status were further adjusted for any residual confounding effects in later multivariate logistic regression analyses.

### 3.2. Association of* ABCG2* Polymorphisms and BC Risk

The frequencies of allelic and genotype distribution for G34A and C421A polymorphism in the* ABCG2* gene and the haplotypes for both BC patients and controls are summarized in Table 1. The frequency distribution of G34A and C421A genotype in control fits well to Hardy-Weinberg equilibrium (*P* = 0.487 and *P* = 0.577, resp.). A significant increased frequency of the G34A GA/AA genotype was observed in BC patients (GA/AA versus GG: *P* = 0.026; adjusted OR: 1.199, 95% CI: 1.022–1.406). The A allele frequency of the G34A polymorphism was significantly different in the BC patients from that in the controls and appeared to be associated with an increased risk of BC (A versus G: *P* = 0.016; adjusted OR: 1.163, 95% CI: 1.028–1.316). Furthermore, the homozygous variant of C421A AA genotype was associated with a higher risk of developing BC (adjusted OR: 1.352, 95% CI: 1.024–1.785; *P* = 0.033). Moreover, the A allele of the C421A polymorphism was associated with a significantly increased risk of BC in comparison to the C allele genotype (adjusted OR: 1.130, 95% CI: 1.001–1.276; *P* = 0.048).

We further investigated the association of the haplotypes of* ABCG2* G34A, C421A with the risk of developing BC. All those frequencies <0.05 will be ignored in the haplotype analysis. We observed that the haplotypes G34A G-C421A C, G34A A-C421A A indicated a lower breast carcinoma risk (adjusted OR: 0.660, 95% CI: 0.589–0.740, *P* = 0.001; adjusted OR: 0.580, 95% CI: 0.465–0.723, *P* < 0.001, resp.). However, the haplotypes G34A A-C421A C, G34A G-C421A A indicated a significantly increased risk for developing BC (adjusted OR: 1.491, 95% CI: 1.300–1.709, *P* = 0.001; adjusted OR: 1.484, 95% CI: 1.299–1.696, *P* < 0.001, resp.) (Table 1).

### 3.3. Correlation of* ABCG2* Polymorphism with Clinicopathological Characteristics

To further assess the clinical utility of* ABCG2* genotyping, we investigated the correlation of G34A and C421A polymorphisms in* ABCG2* gene with clinicopathological features of BC patients by using *χ* ^2^ test and unconditional logistic regression adjusted by age and menopausal status, outlined in Table 2.

We observed that the distribution frequency of* ABCG2* G34A genotype was associated with tumor clinical stages. The frequency (52.9%) of the G34A GG genotype in patients with III or IV tumors was significantly higher than that (43.3%) in patients with I or II tumors (*P* = 0.002, adjusted OR: 0.687, 95% CI: 0.543–0.868). Furthermore,* ABCG2* C421A polymorphism was associated with the ER or PR status in BC patients. The frequency distribution (47.4%) of C421A CC genotype in BC patients with ER positive status was significantly higher than that (39.4%) in patients with ER negative status. Moreover, a higher frequency (48.3%) of the C421A CC genotype was observed in patients with PR positive status compared with patients with PR negative status (Table 2). However, no significant correlation of genotype distributions of* ABCG2* G34A, C421A polymorphisms was observed with other clinicopathological parameters (age at diagnosis, menopausal status, first-degree family history of cancer, tumor size, histology, lymph node metastasis, HER2 status, p53 status, BRCA1 status, and BRCA2 status).

### 3.4. Association between* ABCG2* Polymorphisms and Therapeutic Responses to Neoadjuvant Anthracycline-Based Chemotherapy

In this cohort, 148 patients received neoadjuvant chemotherapy (NCT). Among those patients, 82 patients showed response to NCR and obtained CR and PR. More importantly, a better therapeutic response to anthracycline-based NCT was observed in patients carrying C421A AA homozygote (AA versus CC: *P* = 0.041, adjusted OR = 4.669, 95% CI = 0.826–26.388) (Table 3). No significant association was observed between G34A polymorphism and therapeutic response to anthracycline-based NCT.

### 3.5. Association between* ABCG2* Gene Variants and the Prognosis of BC

To test the hypothesis that* ABCG2* polymorphisms is an independent prognostic factor in this cohort, we further evaluated the correlation of G34A, C421A polymorphisms, and the survival time of the BC patients with postoperative chemotherapies obtained from the Log-rank test and multivariate Cox regression analysis.

**Figure 2:**
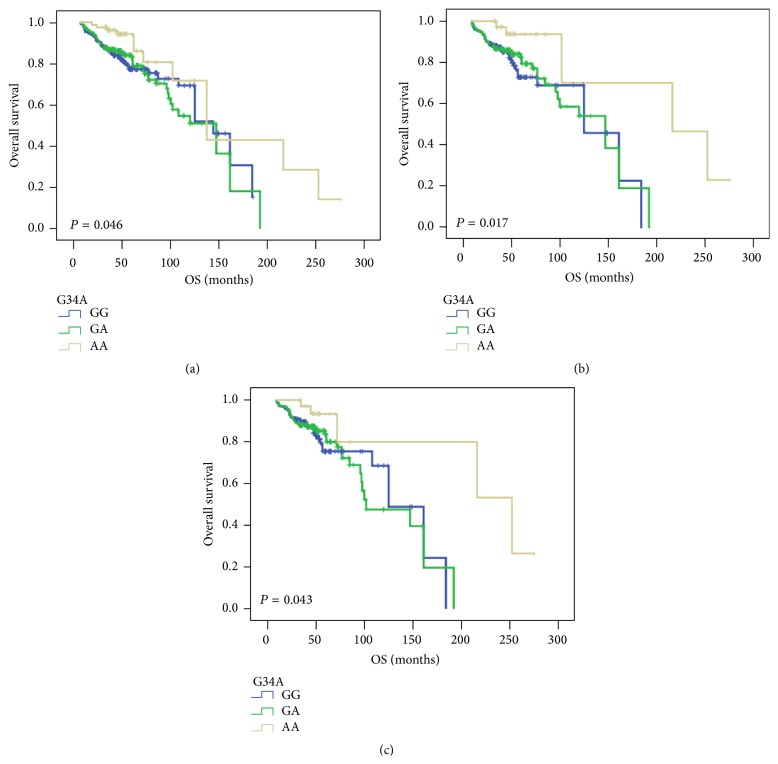
Kaplan-Meier survival curves illustrating the overall survival (OS) in breast cancer patients with the* ABCG2* G34A polymorphism. (a) Patients with anthracycline-based chemotherapy (*n* = 761); (b) patients with ER positive status after anthracycline-based chemotherapy (*n* = 444); (c) patients with PR positive status after anthracycline-based chemotherapy (*n* = 418). Log-rank *P* values were indicated.

We found that* ABCG2* G34A polymorphism had a significant impact on the OS (Log-rank test: *P* = 0.046, Figure 2(a)) in the patients with anthracycline-based chemotherapy (*n* = 761). The estimated median OS for BC patients carrying G34A AA genotype was 166 months (121.45–210.27) compared with the GG genotype carriers' 127 months (112.64–140.19). The multivariate Cox regression analysis further established that carrying G34A AA genotype acted as an independent prognostic factor (adjusted HR: 0.709, 95% CI: 0.507–0.991, *P* = 0.044), outlined in Table 4. Furthermore, a significant association was observed between G34A polymorphism and OS (Log-rank test: *P* = 0.017; Figure 2(b)) in the ER positive patients (*n* = 444). Carrying G34A GA or AA genotype displayed significant effect for prolonged OS compared with those who had GG genotype (147 months, 95% CI: 83.87–166.13 for GA genotype, and 216 months, 95% CI: 68.38–363.62 for AA genotype, versus 125 months, 95% CI: 83.87–166.13 for GG genotype). Moreover, the multivariate Cox regression also verified that G34A AA genotype showed a longer OS (adjusted HR: 0.496, 95% CI: 0.271–0.909, *P* = 0.023). Then, a significant prolonged OS time was observed in G34A polymorphism in PR positive patients treated with anthracycline-based chemotherapy (*n* = 418) (Log-rank test: *P* = 0.043; Figure 2(c)).* ABCG2* G34A AA genotype carriers had a longer OS (252 months, 95% CI: 105.16–398.84, versus 125 months, 95% CI: 94.46–155.54), also verified in multivariate Cox regression analysis (adjusted HR: 0.558, 95% CI: 0.302–1.029, *P* = 0.042). However, there was no impact of* ABCG2* C421A polymorphism observed on DFS and OS in BC patients treated with anthracycline-based chemotherapy.

In addition, multivariate Cox regression analysis identified that tumor size (>2.0 cm), clinical stages (III or IV), and lymph node metastasis status (node positive) of the patients were found to be predictive of worse prognosis in DFS and OS, outlined in Table 4. Meanwhile, the menopausal status of BC patients was observed as a worse DFS (adjusted HR: 1.524, 95% CI: 1.016–2.285, *P* = 0.042) but not OS. The same proportional hazard assumptions were applied for patients with paclitaxel-based chemotherapy (*n* = 79) or anthracycline plus paclitaxel-based chemotherapy (*n* = 101). However, no correlation of* ABCG2* SNPs was observed as a significant predictor of prognosis in patients with paclitaxel- or anthracycline plus paclitaxel-based chemotherapy (Table S2).

## 4. Discussion

ABCG2 was first detected in breast cancer-resistant cells and can facilitate the efflux of a variety of specific endogenous substrates, certain xenobiotics, and anticancer agents (such as Adriamycin/daunorubicin, 7-ethyl-10-hydroxycamptothecin, topotecan, and mitoxantrone), mediating multidrug resistance [7, 24–26]. The polymorphisms of G34A and C421A in the* ABCG2* gene have been reported to alter the expression or activity of* ABCG2* [15, 17, 27, 28] and predispose carriers to a high risk of developing cancers such as nonpapillary renal cell carcinoma [18], DLBCL [21], and hormone-refractory prostate cancer [19]. However, the association between* ABCG2* polymorphisms and BC susceptibility, response to chemotherapy, and prognosis of BC patients still remain to be elucidated. Therefore, the present study attempted to systematically evaluate the association between the* ABCG2* G34A and C421A polymorphisms and the BC susceptibility, clinicopathological features, and clinical outcomes in BC patients after preoperative anthracycline-based NCT or postoperative anthracycline-, paclitaxel-, and anthracycline plus paclitaxel-based chemotherapy.

Little is known regarding the* ABCG2* polymorphism in terms of the potential impacting risk of carcinogenesis and clinical outcomes. In the present study, we found that carriers with* ABCG2* G34A A allele and C421A A allele and haplotype G34A A-C421A C or G34A G-C421A A were significantly associated with the increased BC risk. Our finding that the C421A A allele is associated with susceptibility of BC is consistent with the previous study, which reported that the C421A A allele increased the risk of developing DLBCL [21]. The mechanisms underlying the associations of* ABCG2* polymorphism with susceptibility of carcinogenesis remain unclear. It has been reported that* ABCG2* G34A and C421A variants decrease the expression and the transporter activity of ABCG2 protein [14, 29–31]. Recent paper also showed that individuals carrying heterozygous* ABCG2* variant (C421A) have significantly lower ABCG2 protein expression in their red cells than individuals carrying the wild type [16]. ABCG2 is known to be involved in the efflux of a wide array of structurally divergent substrates such as mitoxantrone, doxorubicin, and topoisomerase I inhibitor [32–34], suggesting that wild* ABCG2* may prevent the cells from accumulation of carcinogens. Therefore, it is likely that the individual with the variants of C421A A allele and/or G34A A allele may have decreased capability to exclude the carcinogens and thereby becomes susceptible to carcinogenesis. However, in contrast to the findings that* ABCG2* C421A A allele conferred an increased risk of BC and DLBCL, Korenaga et al. found that* ABCG2* C421A CC genotype was associated with a higher risk for developing nonpapillary renal cell carcinoma [18]. This difference remains unknown and may result from the distinct molecular mechanisms underlying the development of different carcinomas.

To identify the prognostic markers of multidrug resistance which are targeted effectively to increase chemosensitivity would represent a significant advancement in the treatment of BC. Thereafter, we analyzed the correlation between the* ABCG2* polymorphisms and the prognosis after chemotherapy. As expected, a remarkably better therapeutic response rate (4.669 times) was observed for patients who carried* ABCG2* C421A AA genotype after anthracycline-based NCT. Likewise, similar results also were reported that C421A AA genotype was associated with longer survival of DLBCL in patients younger at diagnose (≤50 years) or with bulky tumor [21]. The increased survival time conferred by* ABCG2* polymorphisms was reported by the findings which identified that* ABCG2* C421A variants decreased the transport activity of substrates such as chemotherapeutic agents (anthracycline) [30, 33, 35]. Accumulation of chemotherapeutic drugs improves the efficacy of chemotherapy in the wild genotype of* ABCG2* gene, thus resulting in a better prognosis. Furthermore, a significantly prolonged OS was observed in all BC patients, ER or PR positive patients with* ABCG2* C421A variants after postoperative anthracycline-based chemotherapy established by Log-rank test and multivariate Cox regression analysis. However, we did not find any statistically significant association between G34A genotypes and the prognosis in BC patients with different chemotherapeutic regimens.

Our results provide evidence that* ABCG2* polymorphisms are associated with BC susceptibility and therapeutic outcome of anthracycline-based NCT and postoperative chemotherapies in a large and well characterized cohort of BC patients; however, several inherent limitations should be considered. Our patient cohort included only Chinese population; therefore, our findings should be confirmed in other ethnic groups or geographic areas in larger samples. Additionally, larger prospective studies or meta-analysis are expected to confirm or compare these results. Nevertheless, the use of the polymorphisms in the* ABCG2* gene may supplement for the current clinical evaluation methods of risk assessment in population studies and perhaps for disease monitoring of BC in the future.

## 5. Conclusions

Our study indicated that* ABCG2* G34A, C421A, and haplotype G34A A–C421A C or G34A G–C421A A were significantly associated with BC risk. The* ABCG2* C421A polymorphism was related to the preoperative neoadjuvant chemotherapeutic response of BC patients, and the* ABCG2* G34A genotypes were associated with the prognostic response in ER positive or PR positive patients with anthracycline-based chemotherapy. Our results suggest that* ABCG2* polymorphism might be a candidate pharmacogenomic factor to assess the BC susceptibility and prognosis in the BC patients with adjuvant chemotherapy.

## Supplementary Material

Supplementary Table S1. Clinicopathological characteristics of the breast carcinoma patients and controls.
Supplementary Table S2. Multivariate COX regression analysis of ABCG2 genetic polymorphisms and patient clinicopathological features in association with DFS and OS in breast carcinoma patients with postoperative paclitaxel-based chemotherapy (n=79), or anthracycline plus paclitaxel-based chemotherapy (n=101).

## Figures and Tables

**Table 1:** Frequency distribution of *ABCG2 *genotypes and their associations with the risk of developing breast carcinoma.

Genotypes	Cases Number (%)	Controls^†^ Number (%)	*P* ^‡^	Adjusted OR (95% CI)^§^
*ABCG2 *G34A				
GG	554 (47.4)	646 (51.9)		1 (reference)
GA	497 (42.5)	494 (39.7)	0.066	1.171 (0.990–1.386)
AA	118 (10.1)	104 (8.4)	0.051	1.329 (0.998–1.770)
GA/AA	615 (52.6)	598 (48.1)	**0.026**	**1.199 (1.022–1.406)**
G allele	1605 (68.65)	1786 (71.8)		1 (reference)
A allele	733 (31.35)	702 (28.2)	**0.016**	**1.163 (1.028–1.316)**
*ABCG2* C421A				
CC	528 (45.2)	598 (48.1)		1 (reference)
CA	509 (43.5)	535 (43.0)	0.397	1.076 (0.909–1.273)
AA	132 (11.3)	111 (8.9)	**0.033**	**1.352 (1.024–1.785)**
CA/AA	641 (54.8)	646 (51.9)	0.155	1.123 (0.975–1.318)
C allele	1565 (66.94)	1731 (69.6)		1 (reference)
A allele	773 (33.06)	757 (30.4)	**0.048**	**1.130 (1.001–1.276)**
Haplotypes				
G34A G-C421A C	967 (41.2)	1281 (51.5)	**0.001**	0.660 (0.589–0.740)
G34A A-C421A C	603 (25.7)	469 (18.8)	**0.001**	**1.491 (1.300–1.709)**
G34A G-C421A A	643 (27.4)	505 (20.3)	**<0.001**	**1.484 (1.299–1.696)**
G34A A-C421A A	133 (5.7)	233 (9.4)	**<0.001**	0.580 (0.465–0.723)

OR indicates odds ratio; CI, confidence interval.

^†^The observed genotype frequency among individuals in the control group was in agreement with Hardy-Weinberg equilibrium (*p* ^2^ + 2*pq* + *q* ^2^ = 1: *χ* ^2^ = 0.483, *P* = 0.487 for *ABCG2* G34A; *χ* ^2^ = 0.378, *P* = 0.577 for *ABCG2* C421A).

^‡^ *P* values were calculated from 2-sided chi-square tests for either genotype distribution or allele frequency.

^§^Adjusted OR and 95% CI values were calculated by unconditional logistic regression adjusted for age and menopausal status.

**Table 2:** Correlations of *ABCG2* polymorphisms with clinicopathological parameters in patients with breast carcinoma.

Characteristic	*ABCG2* G34A				*ABCG2* C421A			
	GG Number(%)	GA/AA Number (%)	*P* ^†,‡^	Adjusted OR (95% CI)^§^	CC Number (%)	CA/AA Number (%)	*P* ^†,‡^	Adjusted OR (95% CI)^§^
Age, yrs								
<50	268 (46.94)	303 (53.06)	0.760^†^	1 (reference)	272 (47.64)	299 (52.36)	0.086^†^	1 (reference)
≥50	286 (47.83)	312 (52.17)	0.445^‡^	1.155 (0.798–1.670)	256 (42.81)	342 (57.19)	0.079^‡^	1.396 (0.963–2.023)
Menopausal status								
Premenopausal	263 (45.90)	310 (54.10)	0.316^†^	1 (reference)	265 (46.25)	308 (53.75)	0.431^†^	1 (reference)
Postmenopausal	291 (48.83)	305 (51.17)	0.222^‡^	0.795 (0.549–1.149)	263 (44.13)	333 (55.87)	0.376^‡^	0.845 (0.583–1.226)
First-degree family history of breast cancer								
No	434 (46.8)	493 (53.2)	0.442^†^	1 (reference)	417 (45.0)	51.0 (55.0)	0.718^†^	1 (reference)
Yes	120 (49.6)	122 (50.4)	0.414^‡^	0.888 (0.669–1.180)	112 (46.3)	130 (53.7)	0.713^‡^	0.948 (0.713–1.260)
Tumor size (cm)								
≤2.0	197 (45.8)	233 (54.2)	0.410^†^	1 (reference)	192 (44.7)	238 (55.3)	0.753^†^	1 (reference)
>2.0	357 (48.3)	382 (51.7)	0.443^‡^	0.911 (0.717–1.157)	337 (45.6)	402 (54.4)	0.712^‡^	0.956 (0.752–1.215)
Histology								
IDC	431 (46.1)	503 (53.9)	0.234^†^	1 (reference)	415 (44.4)	519 (55.6)	0.452^†^	1 (reference)
ILC	36 (52.9)	32 (47.1)	0.286^‡^	0.765 (0.467–1.253)	31 (45.6)	37 (54.4)	0.831^‡^	0.948 (0.577–1.555)
Clinical stages								
I or II	290 (43.3)	380 (56.7)	**0.001** ^†^	1 (reference)	291 (43.4)	379 (56.6)	0.148^†^	1 (reference)
III or IV	264 (52.9)	235 (47.1)	**0.002** ^‡^	**0.687 (0.543–0.868)**	238 (47.7)	261 (52.3)	0.149^‡^	0.842 (0.666–1.064)
Lymph node metastasis status								
Node-negative	291 (46.0)	341 (54.0)	0.317^†^	1 (reference)	283 (44.8)	349 (55.2)	0.724^†^	1 (reference)
Node-positive	263 (49.0)	274 (51.0)	0.363^‡^	0.898 (0.711–1.133)	246 (45.8)	291 (54.2)	0.593^‡^	0.938 (0.743–1.185)
ER status								
Negative	172 (45.1)	209 (54.9)	0.466^†^	1 (reference)	150 (39.4)	231 (60.6)	**0.013** ^†^	1 (reference)
Positive	296 (47.5)	327 (52.5)	0.518^‡^	0.919 (0.710–1.188)	295 (47.4)	328 (52.6)	**0.020** ^‡^	**0.735 (0.566–0.953)**
PR status								
Negative	190 (47.5)	210 (52.5)	0.699^†^	1 (reference)	155 (38.8)	245 (61.2)	**0.003** ^†^	1 (reference)
Positive	278 (46.3)	323 (53.7)	0.663^‡^	1.058 (0.820–1.367)	290 (48.3)	311 (51.7)	**0.004** ^‡^	**0.687 (0.530–0.8902)**
HER2 status								
Negative	233 (46.6)	267 (53.4)	0.945^†^	1 (reference)	222 (44.4)	278 (55.6)	0.828^†^	1 (reference)
Positive	228 (46.8)	259 (53.2)	0.944^‡^	0.991 (0.771–1.273)	216 (44.4)	271 (55.6)	0.999^‡^	1.000 (0.777–1.286)
p53 status								
Negative	158 (46.7)	180 (53.3)	0.828^†^	1 (reference)	142 (42.0)	196 (58.0)	0.155^†^	1 (reference)
Positive	235 (46.0)	276 (54.0)	0.835^‡^	1.030 (0.782–1.357)	240 (47.0)	271 (53.0)	0.147^‡^	0.814 (0.617–1.075)
BRCA1 status								
Negative	65 (48.9)	68 (51.1)	0.645^†^	1 (reference)	55 (41.4)	78 (58.6)	0.626^†^	1 (reference)
Positive	280 (46.7)	320 (53.3)	0.648^‡^	1.092 (0.749–1.590)	262 (43.7)	338 (56.3)	0.590^‡^	0.900 (0.614–1.319)
BRCA2 status								
Negative	141 (49.5)	144 (50.5)	0.354^†^	1 (reference)	118 (41.4)	167 (58.6)	0.406^†^	1 (reference)
Positive	198 (45.9)	233 (54.1)	0.345^‡^	1.156 (0.856–1.560)	192 (44.5)	239 (55.5)	0.443^‡^	0.888 (0.665–1.203)

IDC, invasive ductal carcinoma; ILC, invasive lobular carcinoma; ER, estrogen receptor; PR, progesterone receptor; HER2, human epidermal growth factor receptor; p53, tumor suppressor protein 53; BRCA1, breast carcinoma type 1 susceptibility protein; BRCA2, breast carcinoma type 2 susceptibility protein.

^†^ *P* values were calculated from 2-sided chi-square tests or Fisher's exact test.

^‡^ *P* values were calculated by unconditional logistic regression adjusted for age and menopause state.

^§^Adjusted OR and 95% CI values were calculated by unconditional logistic regression adjusted for age and menopause status.

**Table 3:** Association of *ABCG2* gene polymorphisms with therapeutic response to preoperative neoadjuvant anthracycline-based chemotherapy (*n* = 148).

Variable	Number	RECIST		*P* ^†^	Adjusted OR (95% CI)^‡^
		CR and PR (%)	SD and PD (%)		
*ABCG2 *G34A					
GG	69	40 (58.0)	29 (42.0)		1 (reference)
GA	60	33 (55.0)	27 (45.0)	0.844	0.931 (0.456–1.902)
AA	19	9 (47.4)	10 (52.6)	0.205	0.490 (0.163–1.474)
GA/AA	79	42 (53.2)	37 (46.8)	0.540	1.233 (0.632–2.405)
*ABCG2 *C421A					
CC	70	39 (55.7)	31 (44.3)		1 (reference)
CA	67	35 (52.2)	32 (47.8)	0.771	0.902 (0.449–1.810)
AA	11	8 (72.7)	3 (27.3)	**0.041**	**4.669 (0.826**–**26.388)**
CA/AA	78	43 (55.1)	35 (44.9)	0.798	1.091 (0.559–2.133)

RECIST, Response Evaluation Criteria in Solid Tumors; CR, complete response; PR, partial response; PD, progressive disease; SD, stable disease.

^†^ *P* values were calculated from chi-square tests or Fisher's exact test.

^‡^Adjusted OR and 95% CI values were calculated by unconditional logistic regression adjusted for age and menopause status.

**Table 4:** Multivariate COX regression analysis of *ABCG2 *genetic polymorphisms and patient clinicopathological features in association with DFS and OS in breast carcinoma patients with postoperative anthracycline-based chemotherapy.

Variable	DFS				OS			
	Total *N*	Events *N* (%)	Adjusted HR (95% CI)^†^	*P* ^†^	Total *N*	Events *N* (%)	Adjusted HR (95% CI)^ †^	*P* ^†^
Patients with anthracycline-based chemotherapy								
* ABCG2* G34A								
GG	340	70 (20.6)	1 (reference)		340	70 (20.6)	1 (reference)	
GA	340	72 (21.2)	1.001 (0.718–1.395)	0.997	341	71 (20.9)	0.968 (0.694–1.349)	0.848
AA	81	14 (17.3)	0.777 (0.570–1.059)	0.111	81	12 (14.8)	**0.709 (0.507–0.991)**	**0.044**
* ABCG2* C421A								
CC	367	83 (22.6)	1 (reference)		367	80 (21.8)	1 (reference)	
CA	310	60 (19.4)	0.930 (0.665–1.301)	0.673	311	60 (19.4)	1.010 (0.719–1.418)	0.956
AA	84	13 (15.5)	0.900 (0.706–1.147)	0.395	84	13 (15.5)	0.951 (0.745–1.213)	0.685
ER positive patients with anthracycline-based chemotherapy								
* ABCG2* G34A								
GG	200	43 (21.5)	1 (reference)		200	43 (21.5)	1 (reference)	
GA	204	46 (22.5)	0.977 (0.639–1.492)	0.913	204	45 (22.1)	0.901 (0.591–1.375)	0.630
AA	40	7 (17.5)	0.646 (0.398–1.050)	**0.078**	40	5 (12.5)	**0.496 (0.271–0.909)**	**0.023**
* ABCG2* C421A								
CC	227	54 (23.8)	1 (reference)		227	51 (22.5)	1 (reference)	
CA	163	34 (20.9)	1.098 (0.707–1.705)	0.679	163	34 (20.9)	1.230 (0.786–1.925)	0.364
AA	54	8 (14.8)	0.884 (0.606–1.288)	0.520	54	8 (14.8)	0.927 (0.635–1.355)	0.697
PR positive patients with anthracycline-based chemotherapy								
* ABCG2* G34A								
GG	182	35 (19.2)	1 (reference)		182	35 (19.2)	1 (reference)	
GA	200	43 (21.5)	1.103 (0.699–1.740)	0.674	200	42 (21.0)	1.025 (0.651–1.614)	0.916
AA	36	6 (16.7)	0.643 (0.373–1.110)	0.113	36	5 (13.9)	**0.558 (0.302–1.029)**	**0.042**
* ABCG2* C421A								
CC	216	49 (22.7)	1 (reference)		216	47 (21.8)	1 (reference)	
CA	147	28 (19.0)	1.250 (0.783–1.995)	0.349	147	28 (19.0)	1.260 (0.783–2.027)	0.342
AA	55	7 (12.7)	0.797 (0.534–1.190)	0.267	55	7 (12.7)	0.840 (0.562–1.256)	0.395
Clinicopathological features								
First-degree family history of cancer								
No	927	207 (22.3)	1 (reference)		927	203 (21.9)	1 (reference)	
Yes	242	53 (21.9)	0.899 (0.660–1.223)	0.497	242	51 (21.1)	0.912 (0.668–1.245)	0.562
Tumor size (cm)								
≤2.0	430	79 (18.4)	1 (reference)		430	77 (17.9)	1 (reference)	
>2.0	739	181 (24.5)	**1.494 (1.143–1.953)**	**0.003**	739	177 (18.2)	**1.525 (1.164–1.999)**	**0.002**
Clinical stages								
I or II	670	90 (13.4)	1 (reference)		670	87 (13.0)	1 (reference)	
III or IV	499	170 (34.1)	**3.106 (2.392–4.032)**	**<0.001**	499	167 (33.5)	**3.092 (2.375–4.025)**	**<0.001**
Lymph node metastasis status								
Node-negative	632	94 (14.9)	1 (reference)		632	92 (14.6)	1 (reference)	
Node-positive	537	166 (30.9)	**2.331 (1.800–3.017)**	**<0.001**	537	162 (30.2)	**2.307 (1.778–2.994)**	**<0.001**

HR: hazard ratio; 95% CI, 95% confidence interval; PFS: progression-free survival; RFS: recurrence-free survival; OS: overall survival; reference, reference category; ER, estrogen receptor; PR, progesterone receptor; HER2, human epidermal growth factor receptor; p53, tumor suppressor protein 53; BRCA1, breast carcinoma type 1 susceptibility protein; BRCA2, breast carcinoma type 2 susceptibility protein.

^†^ *P* values and adjusted HR (95% CI) were assessed using multivariate Cox regression analysis adjusted for age and menopause status.

## Abbreviations

*ABCG2*: ATP-binding cassette, subfamily G, member 2
SNPs: Single-nucleotide polymorphisms
BC: Breast carcinoma
IDC: Invasive ductal carcinoma
ILC: Invasive lobular carcinoma
ER: Estrogen receptor
PR: Progesterone receptor
HER2: Human epidermal growth factor receptor
p53: Tumor suppressor protein 53
BRCA1: Breast carcinoma type 1 susceptibility protein
BRCA2: Breast carcinoma type 2 susceptibility protein
OR: Odds ratio
CI: Confidence interval
HR: Hazard ratio
LD: Linkage disequilibrium
PCR-RFLP: Polymerase chain reaction-restriction fragment length polymorphism
HWE: Hardy-Weinberg equilibrium.
