# Supplementary material for: Genetic Variations in ABCG2 Gene Predict Breast Carcinoma Susceptibility and Clinical Outcomes after Treatment with Anthracycline-Based Chemotherapy
Source: Biomed Res Int. 2015 Nov 8;2015:279109. doi: 10.1155/2015/279109 (PMC4655035; doi:10.1155/2015/279109)
Supplement: Supplementary file 1 — Supplementary Table S1. Clinicopathological characteristics of the breast carcinoma patients and controls. Supplementary Table S2. Multivariate COX regression analysis of ABCG2 genetic polymorphisms and patient clinicopathological features in association with DFS and OS in breast carcinoma patients with postoperative paclitaxel-based chemotherapy (n=79), or anthracycline plus paclitaxel-based chemotherapy (n=101). [file 279109.f1.docx]

**Table S1. Clinicopathological characteristics in patients with breast carcinoma and controls**

| **Characteristic** | **Cases** | | **Controls** | | ***P* ^†^** |
| --- | --- | --- | --- | --- | --- |
|  | **No.** | **%** | **No.** | **%** |  |
| **Total no.** | 1169 | 100 | 1244 | 100 |  |
| **Median age [range], y** | 50[22-85] |  | 48[23-70] |  |  |
| **Age, yrs** |  |  |  |  |  |
| <50 | 571 | 48.8 | 589 | 47.35 | 0.953 |
| ≥50 | 598 | 51.2 | 655 | 52.65 |  |
| **Gender** |  |  |  |  |  |
| Women | 1169 | 100 | 1244 | 100 |  |
| **Menopausal status** |  |  |  |  |  |
| Premenopausal | 573 | 49.0 | 597 | 47.99 | 0.321 |
| Postmenopausal | 596 | 51.0 | 647 | 52.01 |  |
| **First-degree family history of cancer** |  |  |  |  |  |
| No | 927 | 79.3 | – | – | – |
| Yes | 242 | 20.7 | – | – |  |
| **Tumor size (cm)** |  |  |  |  |  |
| ≤ 2.0 | 430 | 36.8 | – | – | – |
| >2.0 | 739 | 63.2 | – | – |  |
| **Histology** |  |  |  |  |  |
| IDC | 934 | 79.9 | – | – | – |
| ILC | 68 | 5.8 | – | – |  |
| Others ‡ | 167 | 14.3 | – | – |  |
| **Clinical stages** |  |  |  |  |  |
| I or II | 670 | 57.3 | – | – | – |
| III or IV | 499 | 42.7 | – | – |  |
| **Lymph node metastasis** |  |  |  |  |  |
| Node-negative | 632 | 54.1 | – | – | – |
| Node-positive | 537 | 45.9 | – | – |  |
| Undetermined |  |  |  |  |  |
| **ER status** |  |  |  |  |  |
| Negative | 381 | 32.6 | – | – | – |
| Positive | 623 | 53.3 | – | – |  |
| Undetermined | 165 | 14.1 | – | – |  |
| **PR status** |  |  |  |  |  |
| Negative | 400 | 34.2 | – | – | – |
| Positive | 601 | 51.4 | – | – |  |
| Undetermined | 168 | 14.4 | – | – |  |
| **HER2 status** |  |  |  |  |  |
| Negative | 500 | 42.8 | – | – | – |
| Positive | 487 | 41.7 | – | – |  |
| Undetermined | 182 | 15.6 | – | – |  |
| **p53 status** |  |  |  |  |  |
| Negative | 338 | 28.9 | – | – | – |
| Positive | 511 | 43.7 | – | – |  |
| Undetermined | 320 | 27.4 | – | – |  |
| **BRCA1 status** |  |  |  |  |  |
| Negative | 133 | 11.4 | – | – | – |
| Positive | 600 | 51.3 | – | – |  |
| Undetermined | 436 | 37.3 | – | – |  |
| **BRCA2 status** |  |  |  |  |  |
| Negative | 285 | 24.4 | – | – | – |
| Positive | 431 | 36.9 | – | – |  |
| Undetermined | 453 | 38.8 | – | – |  |
| **Preoperative neoadjuvant chemotherapy** |  |  |  |  |  |
| Anthracycline-based chemotherapy | 148 | 12.66 | – | – | – |
| **Postoperative therapeutic regimens** |  |  |  |  |  |
| Anthracycline-based chemotherapy | 761 | 65.1 | – | – | – |
| Paclitaxel-based chemotherapy | 79 | 6.8 | – | – |  |
| Anthracycline plus Paclitaxel-based chemotherapy | 101 | 8.6 | – | – |  |
| Other chemotherapies or treatments | 228 | 19.5 | – | – |  |

^†^ Determined using a 2–sided chi–square test.

^‡^Others contains: invasive cribriform carcinoma, medullary carcinoma, and invasive papillary carcinoma.

**Table S2. Multivariate COX regression analysis of *ABCG2* genetic polymorphisms and patient clinicopathological features in association with DFS and OS in breast carcinoma patients with postoperative paclitaxel-based chemotherapy (*n*=79), or anthracycline plus paclitaxel-based chemotherapy (*n*=101)**

| **Variable** | **DFS** | | | | **OS** | | | |
| --- | --- | --- | --- | --- | --- | --- | --- | --- |
|  | **Total**  **N** | **Events**  **N (%)** | **Adjusted**  **HR(95%CI)** ^†^ | ***P*** ^†^ | **Total**  **N** | **Events**  **N (%)** | **Adjusted**  **HR(95%CI)** ^†^ | ***P*** ^†^ |
| **Patients with paclitaxel-based chemotherapy** | | | |  |  |  |  |  |
| ***ABCG2* rs2231137** |  |  |  |  |  |  |  |  |
| GG | 42 | 12(28.6) | 1 (reference) |  | 42 | 12(28.6) | 1 (reference) |  |
| GA | 29 | 9(31.0) | 1.117(0.458-2.725) | 0.808 | 29 | 9(31.0) | 1.143(0.467-2.796) | 0.770 |
| AA | 8 | 5(62.5) | 1.563(0.912-2.678) | 0.104 | 8 | 5(62.5) | 1.383(0.805-2.374) | 0.240 |
| ***ABCG2* rs2231142** |  |  |  |  |  |  |  |  |
| CC | 30 | 10(33.3) | 1 (reference) |  | 30 | 10(33.3) | 1 (reference) |  |
| CA | 40 | 13(32.5) | 0.832(0.357-1.936) | 0.669 | 40 | 13(32.5) | 0.709(0.298-1.685) | 0.436 |
| AA | 9 | 3(33.3) | 1.024(0.527-1.993) | 0.943 | 9 | 3(33.3) | 0.891(0.451-1.761) | 0.740 |
| **Patients with paclitaxel-, or anthracycline plus paclitaxel-based chemotherapy** | | | | | |  |  |  |
| ***ABCG2* rs2231137** |  |  |  |  |  |  |  |  |
| GG | 49 | 15(30.6) | 1 (reference) |  | 49 | 14(28.6) | 1 (reference) |  |
| GA | 41 | 18(43.9) | 1.456(0.696-3.046) | 0.318 | 41 | 17(41.5) | 1.423(0.678-2.987) | 0.351 |
| AA | 11 | 5(45.5) | 1.116(0.587-2.122) | 0.737 | 11 | 5(45.5) | 1.113(0.585-2.119) | 0.744 |
| ***ABCG2* rs2231142** |  |  |  |  |  |  |  |  |
| CC | 40 | 17(42.5) | 1 (reference) |  | 40 | 16(40.0) | 1 (reference) |  |
| CA | 51 | 19(37.30 | 0.809(0.401-1.630) | 0.553 | 51 | 18(35.3) | 0.839(0.414-1.698) | 0.625 |
| AA | 10 | 2(20.0) | 0.756(0.359-1.594) | 0.463 | 10 | 2(20.0) | 0.774(0.767-1.633) | 0.501 |
| **Clinicopathological features** | | |  |  |  |  |  |  |
| **ER status** |  |  |  |  |  |  |  |  |
| Negative | 381 | 74(19.4) | 1 (reference) |  | 381 | 75(19.7) | 1 (reference) |  |
| Positive | 623 | 151(24.2) | 1.254(0.947-1.662) | 0.115 | 623 | 145(23.3) | 1.164(0.878-1.545) | 0.291 |
| **PR status** |  |  |  |  |  |  |  |  |
| Negative | 400 | 83(20.8) | 1 (reference) |  | 400 | 81(20.2) | 1 (reference) |  |
| Positive | 601 | 139(23.1) | 1.111(0.842-1.465) | 0.457 | 601 | 136(22.6) | 1.092(0.825-1.445) | 0.538 |
| **Her2 status** |  |  |  |  |  |  |  |  |
| Negative | 500 | 116(23.2) | 1 (reference) |  | 500 | 113(22.6) | 1 (reference) |  |
| Positive | 487 | 101(20.7) | 0.892(0.681-1.169) | 0.408 | 487 | 99(20.3) | 0.900(0.685-1.181) | 0.447 |
| **p53 status** |  |  |  |  |  |  |  |  |
| Negative | 340 | 70(20.6) | 1 (reference) |  | 340 | 67(19.7) | 1 (reference) |  |
| Positive | 511 | 117(22.9) | 1.099(0.815-1.482) | 0.537 | 511 | 116(22.7) | 1.147(0.847-1.553) | 0.376 |
| **BRCA1 status** |  |  |  |  |  |  |  |  |
| Negative | 133 | 23(17.3) | 1 (reference) |  | 133 | 23(17.3) | 1 (reference) |  |
| Positive | 600 | 135(22.5) | 1.236(0.792-1.928) | 0.351 | 600 | 131(21.8) | 1.235(0.791-1.928) | 0.353 |
| **BRCA2 status** |  |  |  |  |  |  |  |  |
| Negative | 285 | 54(18.9) | 1 (reference) |  | 285 | 54(18.9) | 1 (reference) |  |

^†^*P* values, Adjusted HR (95%CI) were assessed using multivariate Cox regression analysis adjusted for age, menopause status.
